# Diestrous Ovulations in Pregnant Mares as a Response to Low Early Postovulatory Progestogen Concentration

**DOI:** 10.3390/ani10122249

**Published:** 2020-11-30

**Authors:** Carolina T. C. Okada, Martim Kaps, Javier Perez Quesada, Camille Gautier, Jörg Aurich, Christine Aurich

**Affiliations:** 1 Platform Artificial Insemination and Embryo Transfer, Department for Small Animals and Horses, Vetmeduni Vienna, Veterinärplatz 1, 1210 Vienna, Austria; okadac@staff.vetmeduni.ac.at (C.T.C.O.); Martim.Kaps@vetmeduni.ac.at (M.K.); perezquesadajavier@gmail.com (J.P.Q.); CamilleMarie.Gautier@vetmeduni.ac.at (C.G.); 2 Section for Obstetrics, Gynaecology and Andrology, Department for Small Animals and Horses, Vetmeduni Vienna, Veterinärplatz 1, 1210 Vienna, Austria; joerg.aurich@vetmeduni.ac.at

**Keywords:** corpus luteum, early pregnancy in mares, hypothalamic-pituitary axis, luteal phase ovulation, negative feedback, progesterone

## Abstract

**Simple Summary:**

During early pregnancy in mares, progestogen is synthesized by the primary corpus luteum, which is the only source of progestogen until endometrial cup and accessory corpus luteum formation, from day 36 of pregnancy onwards. In the present study, we investigated the hormonal profile (gonadotrophin and progestogen concentrations) of 11 mares after experimental reduction of primary corpus luteum function. Two pregnancies of each mare were assigned to the control and treatment groups, respectively, and were analyzed until day 34. Low plasma progestogen concentration caused by the treatment reduced the negative feedback on the hypothalamic-pituitary axis, stimulating gonadotropin release, and luteal tissue response. Progestogen concentration restoration soon after treatment suggests a rebound effect and the resurgence of luteal function. In addition, diestrous ovulation was observed between days 11 and 15 in five treatment pregnancies (5/11), but none of the controls (0/11). Although the total luteal area increased after diestrous ovulations, corpus luteum size was not correlated to progestogen secretion. Results suggest that diestrous ovulations during early pregnancy in mares may reflect low progestogen concentrations in the early postovulatory period.

**Abstract:**

Spontaneous prolongation of the luteal phase has been described in horses, but the underlying causes are still unclear. The present study investigated details of gonadotrophin and progestogen secretion in pregnant mares (n = 11) with or without experimentally reduced early postovulatory luteal function. From days 0 to 3 after ovulation, they were treated with the prostaglandin F_2α_ (PGF_2α_) analogue cloprostenol or left untreated. After conceptus collection on day 34, they were assigned to the opposite treatment. Mares were affiliated to the group primary corpus luteum (n = 6) or diestrous corpus luteum (n = 5) depending on diestrous corpus luteum (CL) detection in the PGF pregnancy. For statistical comparisons, a *p*-value < 0.05 was significant. There was an effect of treatment (*p* < 0.01), but not of group on progestogen concentration. The concentration of LH was higher in PGF-treated than in untreated pregnancies (*p* < 0.05), but did not differ between groups. The FSH concentration did not differ between groups nor treatments. The total luteal tissue area was greater in mares with a diestrous ovulation during the PGF treatment pregnancy. Low progestogen concentration in the early postovulatory phase diminish the negative feedback on the hypothalamic-pituitary axis in early pregnancy and, thus, stimulate a luteal tissue response. Detection of secondary CL at the time of pregnancy examination in mares may reflect that early post-ovulatory progestogen concentrations were low.

## 1. Introduction

In most animal species, prolongation of the luteal phase in the absence of pregnancy occurs only if experimentally induced or associated with uterine pathologies (for review see [1]). In horses, however, spontaneous prolongation of the luteal phase for periods of two to three months is not uncommon [1,2,3,4]. An association of this phenomenon with diestrous ovulations in at least part of the affected horses was suggested [1] and later on confirmed [4]. In approximately 10% of mares, luteal phase ovulations, i.e., ovulations occurring more than three days after a follicular phase ovulation, were detected in pregnant as well as non-pregnant mares [4]. These diestrous ovulations may occur as early as day 4 or 5, but also at later times after the follicular phase ovulation [4]. If diestrous ovulations occur later than day 9, they will, however, result in prolonged luteal activity [2,3,4], because of non-responsiveness of the still immature luteal tissue at the time of endometrial prostaglandin release, although the primary corpus luteum may undergo luteolysis [3,4].

The underlying causes for the occurrence of luteal phase ovulations in horses are still largely unclear. An association with low progesterone concentrations has been suggested [4]. There is, however, evidence that low postovulatory progesterone concentrations in mares may be also associated with increased pregnancy loss [5,6,7]. In several species, the concentration of plasma progesterone during the first days after ovulation substantially influences endometrial function with regard to conceptus nutrient supply [8] because it induces downregulation of endometrial progesterone receptors. Timely progesterone receptor downregulation is an important step in the preparation of the endometrium for early pregnancy and placentation [9,10]. This has also been demonstrated in horses [5,7,11]. 

Treatment of mares with PGF_2α_ and its analogues during the early postovulatory phase does not induce complete luteolysis, but results in markedly reduced plasma progestogen concentrations, followed by a steady progestogen increase reflecting a certain resumption of luteal function [5,7,12,13,14,15]. We could recently demonstrate that the experimentally reduced early postovulatory progestogen concentration in pregnant mares was not only associated with delayed conceptus development, but also with an increased incidence of luteal phase ovulations between days 11 and 15 after ovulation [7]. We therefore hypothesized that the reduced plasma progestogen concentrations due to a considerable reduction of the negative feedback on the pituitary [16,17] allow for increased gonadotrophin release and subsequent ovulation. The detection of secondary ovulations at pregnancy examination may therefore suggest low progestogen concentrations during the early postovulatory phase, and thus be indicative of an increased risk for the pregnancy. In the present study, details of gonadotrophin and progestogen release together in pregnant mares with or without experimentally reduced early postovulatory luteal function [7] were investigated. 

## 2. Material and Methods

### 2.1. Experimental Animals

Eleven healthy Haflinger mares with proven fertility were available for the study. They were between 4 and 11 years old (5.7 ± 2.4) and weighed 456 ± 7.5 kg. Mares were maintained as one herd in a large outdoor paddock and a covered shed, they were fed hay twice a day; water was available ad libitum. Before the experiment, a complete breeding soundness evaluation was performed, including uterine bacteriological analysis and histopathology. Mares enrolled into the study presented a histological classification of the endometrium of I or IIA [18]. The study was approved by the Austrian Federal Ministry for Science and Research (animal experimentation license 68.205/067-V/3b/2018). 

### 2.2. Experimental Design and Management of Mares

The mares were submitted to regular examinations of the reproductive tract by transrectal palpation and ultrasonography (DP-6600Vet, Mindray, Shenzhen, China) in order to monitor the estrus. When a dominant follicle >35 mm in diameter was detected along with uterine edema, mares were inseminated with raw semen (500 × 10^6^ motile spermatozoa) from a fertile stallion at 48-h intervals until spontaneous ovulation (disappearance of the preovulatory follicle) occurred. On the ovulation day (day 0), the mares were randomly assigned to treatment (PGF) with the prostaglandin F2α (PGF_2α_) analogue cloprostenol (125 µg once daily; Estrumate, MSD, Vienna, Austria) from day 0 until day 3 or left untreated as control (CON), as previously described [5,7]. 

Pregnancy diagnosis was performed on day 10 by transrectal ultrasonographic examination. Conceptus growth and development as well as ovarian function was evaluated daily until 24 days, then on days 29 and 34 as described [7]. After conceptus collection on day 34 as previously reported [7], mares rested one estrous cycle before assigned to the opposite treatment, serving as their own control.

When the mare was confirmed pregnant on day 10, the luteal tissue area was evaluated by transrectal ultrasonography once daily until day 24 and on days 29 and 34. At the same time, ovaries were scanned by transrectal ultrasound for follicular growth. The presence of follicles of preovulatory size (i.e., ≥30 mm in diameter) and their disappearance (=ovulation) was noted. Luteal phase (i.e., diestrus) ovulations were detected in 5 out of 11 treatment pregnancies, but in none of the control pregnancies. One diestrous ovulation was detected in each of these treatment pregnancies and occurred on days 11, 12, 13, 14, and 15 (day 0 = day of ovulation). Depending on the detection of a diestrous ovulation after day 9 of the treatment pregnancy, mares were affiliated to either the group primary CL (n = 6) or the group diestrous CL (n = 5). No additional diestrous ovulations were detected. 

Blood was collected by venipuncture of one jugular vein into heparinized tubes (Vacuette, Greiner, Kremsmünster, Austria) once daily from 1 day before ovulation until 14 days post ovulation and on days 19, 24, 29, and 34. The plasma was separated by centrifugation at 1200× *g* for 10 min and stored at −20 °C until analysis. 

### 2.3. Determination of the Total Luteal Tissue Area

The CL area was analyzed by software ImageJ (National Institutes of Health, Bethesda, MD, USA) with previous scale bar setting in order to avoid dimensional differences. With the images of the CL in maximal diameter, a line was drawn manually between luteal tissue and surroundings, calculating the cross-sectional area of the CL. In case of a diestrous ovulation, the luteal tissue area was calculated in the same way and added to the diameter of the primary CL. Values are given as total luteal tissue area, i.e., the area of the primary CL plus—in mares with a diestrous ovulation—the area of the diestrous CL. 

### 2.4. Hormone Assays

Plasma progestogen concentration was evaluated by enzyme-linked immunosorbent assay (Enzo Progesterone ELISA, Cat. No.: ADI-901-011, Enzo Life Sciences, Farmingdale, NY, USA) as described [5]. Before analysis, plasma was diluted 1:100. The intra-assay coefficient of variation was 7.5%, the inter-assay coefficient of variation was 15.3%, and the minimal detectable concentration 4.9 pg/mL.

Plasma LH analysis was performed by radioimmunoassay (RIA) previously validated for equine samples [19]. For standard and iodinated samples, highly purified pituitary derived equine LH was selected (Roser 2001B; University of California, Davis, CA, USA) and was used as described [20] with modifications [21]. The sensitivity of the assay was 0.25 ng/mL and the intra- and inter-assay coefficients of variation were both <10%. 

Similarly, FSH concentration was analyzed by a validated RIA [19] with minor modifications [21]. For standards and iodination, highly purified pituitary derived equine FSH (e265B, Dr. H. Papkoff, University of California, Davis, CA, USA) was selected. The primary antibody (3D-2 anti-oFSH, used at 1:20,000 dilution) was acquired from Dr. D.L. Thompson (Louisiana State University, Baton Rouge LA, USA). The sensitivity of the assay was 0.5 ng/mL and the intra- and inter-assay coefficients of variation were 6.25% and 6.5%, respectively. 

### 2.5. Statistical Analysis

Statistical analysis was performed with the IBM SPSS statistics software (version 24.0; Armonk, NY, USA). Data were tested for normal distribution by Kolmogorov-Smirnov test. Endpoints that were determined repeatedly were compared by General Linear Model ANOVA for repeated measures (concentration of plasma progestogens, LH, and FSH, luteal tissue area) with group (with vs. without diestrous ovulation) as between subject factor and pregnancy (treatment vs. control) and time (day after ovulation) as within subject factors. The number of mares with presence of a follicle of preovulatory size between days 10 and 34 were compared by non-parametric Kruskal–Wallis test followed by Mann–Whitney test in case of significant differences. Correlations were analyzed using the Spearman–Rho correlation test. For all statistical comparisons, a *p*-value < 0.05 was considered significant. Values are given as mean ± SEM if not stated otherwise.

## 3. Results

**Figure 1:**
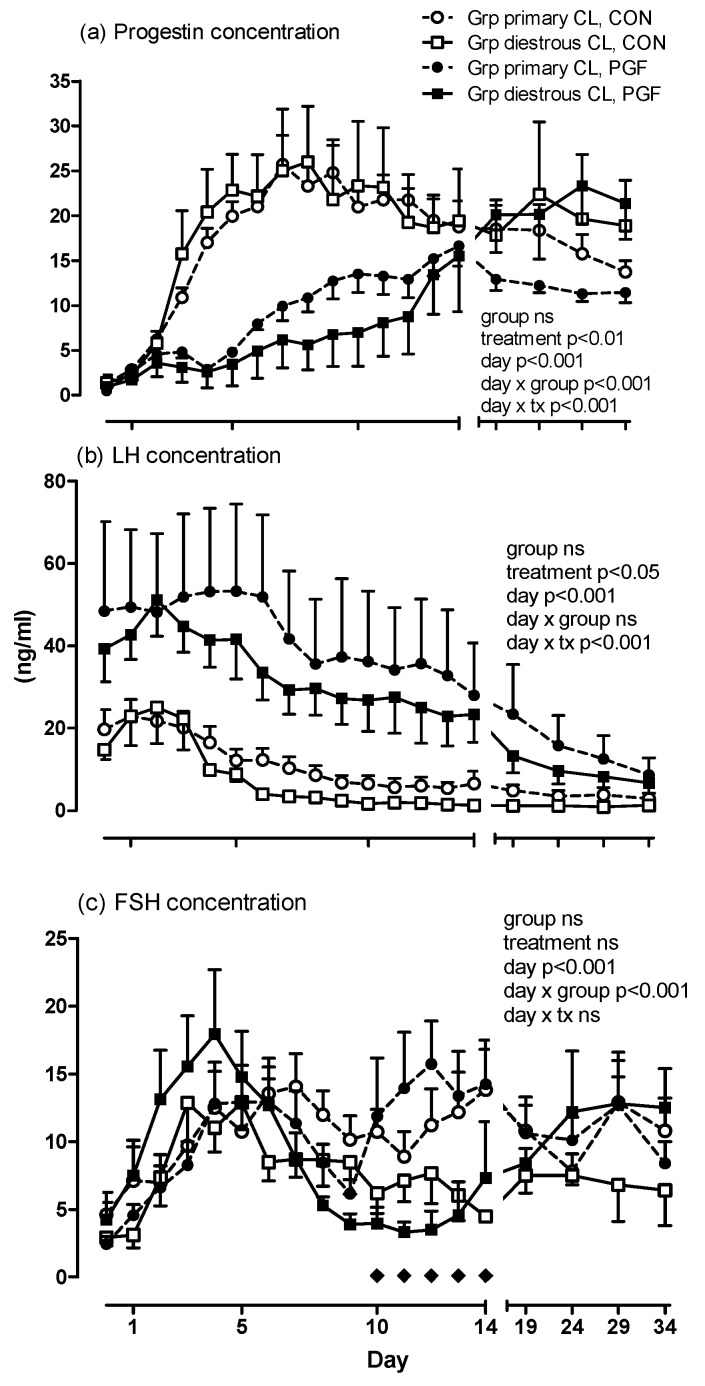
Concentrations of (**a**) progestogens, (**b**) LH and (**c**) FSH in control (CON) and prostaglandin F (PGF) pregnancies with mares grouped by the absence (Group [Grp] primary corpus luteum [CL]; n = 6) or presence (Grp diestrous CL; n = 5) of a diestrous ovulation in the PGF pregnancy. For group legends see Figure a. In Figure c, diamonds indicate days with diestrous ovulations in PGF pregnancies.

There was an effect of treatment (early postovulatory PGF vs. control; *p* < 0.01), but not of group (with or without luteal phase ovulation) on plasma progestogen concentration until day 34 of pregnancy (Figure 1a). There was an interaction of treatment with day (*p* < 0.001) as well as group with day (*p* < 0.001). In both groups during treatment pregnancies, progestogen concentration rose steadily after day 3, but this increase was more pronounced in the group of mares with luteal phase ovulations. There was no correlation between progestogen concentrations determined on days 1 to 10 and the interval from estrous ovulation to diestrous ovulation. 

The concentration of plasma LH was higher in PGF-treated than in untreated pregnancies (*p* < 0.05; Figure 1b). The plasma LH concentration steadily decreased over time (*p* < 0.001) with slight differences in the LH concentration pattern from days 0 to 34. Group (with or without luteal phase ovulations in the treatment pregnancy) did not affect plasma LH concentrations (group and day × group: n.s.). The concentration of plasma FSH did not differ between groups nor treatments (Figure 1c). There was, however, an interaction between group and day with the group of mares with luteal phase ovulations having a different FSH secretion pattern than mares without luteal phase ovulations independent of treatment.

**Figure 2:**
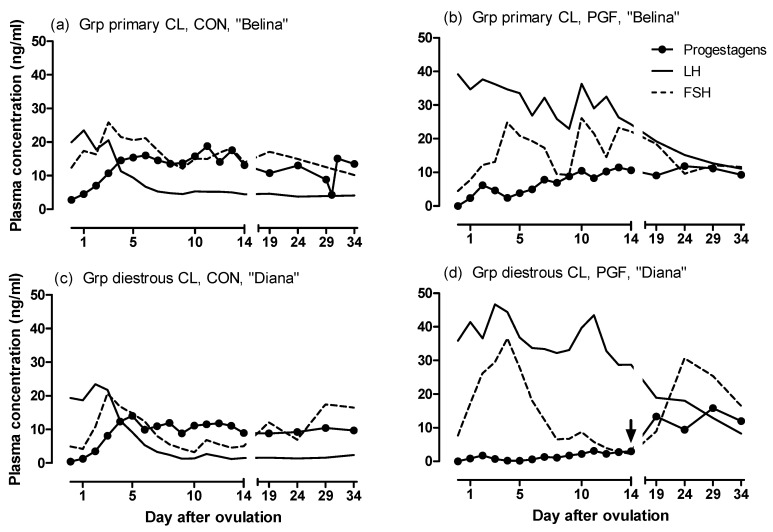
Concentrations of progestogens, LH and FSH in one representative mare from the group with primary CL in the (**a**) CON and (**b**) PGF pregnancy, and from the group with diestrous ovulation in the PGF pregnancy in her (**c**) CON and (**d**) PGF pregnancy. The day of the diestrous ovulation in (**d**) is indicated by an arrow.

Individual patterns of the plasma concentrations of progestogens, LH and FSH in one representative mare from each group are shown in Figure 2. 

**Figure 3:**
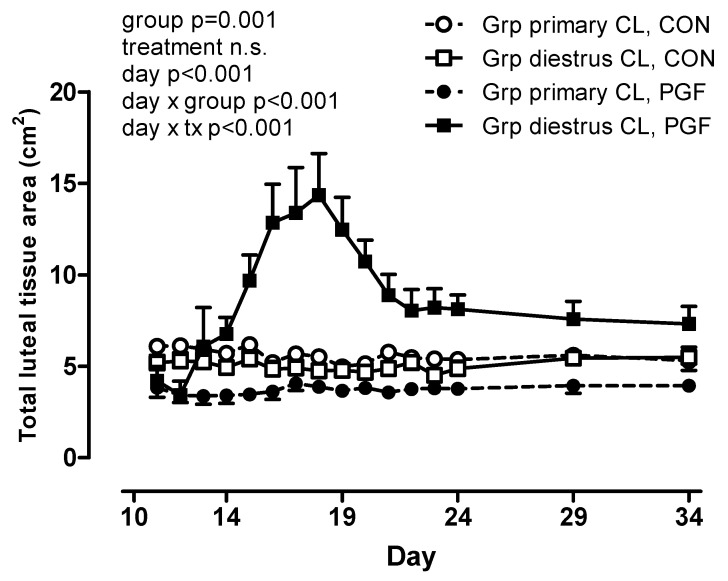
Total luteal tissue area (cm^2^) in CON and PGF pregnancies with mares grouped by the absence (Grp primary CL; n = 6) or presence (Grp diestrous CL; n = 5) of a diestrous ovulation in the PGF pregnancy.

The total luteal tissue area was affected by group (Figure 3) with a larger total luteal tissue area in mares with a diestrous ovulation during the PGF-treatment pregnancy (group *p* = 0.001, treatment n.s., day × group *p* < 0.001, day × treatment *p* < 0.001). No significant correlations between luteal tissue area and progestogen concentrations were determined. 

Early postovulatory PGF treatment increased the number of mares with ovarian follicles of preovulatory size on days 11 and 12 (*p* < 0.05) but not on any other days of the study (e.g., number of mares with preovulatory follicles on day 11: Group primary CL, CON: 0/6, Group diestrous CL, CON: 0/5, Group primary CL, PGF: 2/6, Group diestrous CL, PGF: 4/5). 

## 4. Discussion

In the present study, plasma progestogen concentration in pregnant mares treated with a PGF analogue between days 0 and 3 after ovulation was lower than in untreated control pregnancies, but steadily increased from day 4 after ovulation. This suggests the resurgence of functional luteal tissue and a progestogen rebound effect starting soon after PGF treatment. Such effects were already described previously not only in horses [14], but also in cows [22,23]. Based on the results of the present investigation it can be concluded that the increase in progestogen concentration after PGF treatment is possible because of recovery of luteal function in some mares, but also includes diestrous ovulations in other mares. Both events most likely depend on the higher concentration of plasma LH in PGF treated mares that was detected from day 0 after ovulation onwards.

Luteal tissue resumption in PGF-treated mares that did not experience a diestrous ovulation is possible because of the presence of LH receptors in equine luteal tissue [24] together with a high mitotic activity of progesterone producing luteal cells in the early luteal phase [25]. Similarly, progesterone concentrations in untreated mares could be stimulated when they were treated with the LH analogue hCG (human chorionic gonadotrophin) on days 3, 4, and 5 after ovulation [24], but not thereafter [26]. The luteal phase ovulation detected in five of the 11 mares in the PGF pregnancies of the present study was even more effective in increasing plasma progestogen concentrations. The increase in plasma LH concentration in PGF-treated mares of the present study occurred thus in time to allow for a luteal tissue response. The response was, however, not in good time to avoid a delayed development of the conceptus [7]. The present results thus suggest that the detection of secondary corpora lutea together with a viable conceptus at the time of pregnancy examination in mares may reflect that early post-ovulatory progestogen concentrations were low and the respective pregnancy may be more at risk of undergoing early embryonic death. Embryo survival is maintained at plasma progesterone concentrations >2.5 ng/mL [27]. Results from the present study together with the finding of a delayed conceptus development in progestogen-deprived mares [9], however, support the idea of progestin supplementation in mares with low early postovulatory plasma progestogen concentration to ensure adequate conceptus development. This idea is further supported by the following findings: (i) the use of ovulation inducing agents in the estrus used for breeding reduced the incidence of early pregnancy loss in Thoroughbred mares [6]; (ii) the crown rump-length of embryos/fetuses that underwent early pregnancy loss was smaller than from ongoing pregnancies [28]; (iii) treatment of estrous mares with ovulation-inducing agents did not only stimulate ovulation, but also luteal function, progestogen production, and conceptus development [26]. 

The higher plasma LH concentration in PGF-treated in comparison to control pregnancies is most probably an effect of the low progestogen concentration and the related reduction of the negative feedback of progestogens on pituitary LH secretion [16,29]. In agreement with this suggestion, ovulations in mares with progesterone releasing intravaginal device have been described and were associated with high plasma LH concentrations [30]. In the mare, progestogen synthesis in luteal tissue starts already at a very early time of CL formation and thus circulating concentrations of progestogens immediately increase at the time of ovulation [31]. In the present study, treatment with the PGF analogue was initiated at detection of disappearance of the preovulatory follicle, and, therefore, would have immediately affected luteal tissue formation. Differences in progestogen concentrations between PGF-treated and untreated mares are, thus, likely to result also in very early differences in the luteal feedback on pituitary function. As an alternative cause of increased LH concentrations at the beginning of the luteal phase, direct pituitary effects of the PGF analogue [32,33,34], have to be considered. Interestingly, no effects of PGF treatment on plasma FSH concentrations were detected in the present study. Although a strong association between pulses of GnRH, LH, and FSH has been demonstrated in mares [35], differences in LH and FSH release in response to the reduced plasma progestogen concentration in this study are likely to depend on factors that regulate LH and FSH secretion independent from GnRH as for example kisspeptin [36,37]. 

In the present investigation, the luteal tissue area of the primary corpus luteum determined from days 11 to 34 after ovulation was stable and not influenced by PGF treatment. Diestrous ovulation, however, considerably increased the total luteal tissue area. This increase was not reflected in plasma progestogen concentration. This confirms that corpus luteum size in the mare is not indicative of progestogen secretion [26]. Plasma progestogen concentration alone is, therefore, not sufficient to characterize luteal function in the mare. The complementary assessment of luteotrophic factors, such as growth factors, cytokines and, most importantly, prostaglandins, has to be considered [38]. 

## 5. Conclusions

Low progestogen concentration induced by repeated treatment with a PGF_2α_ analog in the first days after ovulation affected the negative feedback on the hypothalamic-pituitary axis in early pregnant mares and stimulated a luteal tissue response that was, however, not successful to allow for normal embryo development. The results suggest that the detection of secondary corpora lutea at the time of pregnancy examination in mares may reflect that early post-ovulatory progestogen concentrations were low and the respective pregnancy may be more at risk to undergo early embryonic death.
